# Inhibition of Toxic Shock Syndrome-Associated *Staphylococcus aureus* by Probiotic Lactobacilli

**DOI:** 10.1128/spectrum.01735-23

**Published:** 2023-07-05

**Authors:** Patrick M. Schlievert, Adriana V. Gaitán, Samuel H. Kilgore, Amy L. Roe, Johanna Maukonen, Liisa Lehtoranta, Donald Y. M. Leung, Daniel S. Marsman

**Affiliations:** a Department of Microbiology and Immunology, University of Iowa; Carver College of Medicine, Iowa City, Iowa, USA; b The Procter & Gamble Company, Cincinnati, Ohio, USA; c IFF Health & Biosciences, Kantvik, Finland; d Department of Pediatrics, National Jewish Health, Denver, Colorado, USA; Emory University School of Medicine

**Keywords:** *Staphylococcus aureus*, lactobacilli, probiotic, superantigens, toxic shock syndrome toxin-1, two-component system

## Abstract

Staphylococcus aureus is a human pathogen with many infections originating on mucosal surfaces. One common group of S. aureus is the USA200 (CC30) clonal group, which produces toxic shock syndrome toxin-1 (TSST-1). Many USA200 infections occur on mucosal surfaces, particularly in the vagina and gastrointestinal tract. This allows these organisms to cause cases of menstrual TSS and enterocolitis. The current study examined the ability of two lactobacilli, Lactobacillus acidophilus strain LA-14 and Lacticaseibacillus rhamnosus strain HN001, for their ability to inhibit the growth of TSST-1 positive S. aureus, the production of TSST-1, and the ability of TSST-1 to induce pro-inflammatory chemokines from human vaginal epithelial cells (HVECs). In competition growth experiments, L. rhamnosus did not affect the growth of TSS S. aureus but did inhibit the production of TSST-1; this effect was partially due to acidification of the growth medium. L. acidophilus was both bactericidal and prevented the production of TSST-1 by S. aureus. This effect appeared to be partially due to acidification of the growth medium, production of H_2_O_2_, and production of other antibacterial molecules. When both organisms were incubated with S. aureus, the effect of L. acidophilus LA-14 dominated. In *in vitro* experiments with HVECs, neither lactobacillus induced significant production of the chemokine interleukin-8, whereas TSST-1 did induce production of the chemokine. When the lactobacilli were incubated with HVECs in the presence of TSST-1, the lactobacilli reduced chemokine production. These data suggest that these two bacteria in probiotics could reduce the incidence of menstrual and enterocolitis-associated TSS.

**IMPORTANCE** Toxic shock syndrome (TSS) Staphylococcus aureus commonly colonize mucosal surfaces, giving them the ability to cause TSS through the action of TSS toxin-1 (TSST-1). This study examined the ability of two probiotic lactobacilli to inhibit S. aureus growth and TSST-1 production, and the reduction of pro-inflammatory chemokine production by TSST-1. Lacticaseibacillus rhamnosus strain HN001 inhibited TSST-1 production due to acid production but did not affect S. aureus growth. Lactobacillus acidophilus strain LA-14 was bactericidal against S. aureus, partially due to acid and H_2_O_2_ production, and consequently also inhibited TSST-1 production. Neither lactobacillus induced the production of pro-inflammatory chemokines by human vaginal epithelial cells, and both inhibited chemokine production by TSST-1. These data suggest that the two probiotics could reduce the incidence of mucosa-associated TSS, including menstrual TSS and cases originating as enterocolitis.

## INTRODUCTION

Staphylococcus aureus is a ubiquitous pathogen that most often originates from mucosal surfaces but can also cause infections across skin barriers (1, 2). The CDC classifies S. aureus strains as USA100 to -1100 based on pulsed-field gel electrophoresis (3). Among these clonal groups is the USA200 (CC30) clonal group. USA200 strains of S. aureus, present on mucosal surfaces, have unique properties compared to other clonal groups. USA200 S. aureus produces the superantigen toxic shock syndrome toxin-1 (TSST-1), produces β-cytotoxin (the hot-cold hemolysin), and has greatly reduced alpha-toxin production compared to other clonal groups (2, 4–6). This combination of virulence factors appears to restrict the group to mucosal surfaces or damaged skin (2, 5, 6).

TSST-1 is the cause of 100% of menstrual TSS cases from human vaginal USA200 S. aureus (2, 7–9). Additionally, a few cases of enterocolitis are associated with intestinal infection with TSST-1 USA200 S. aureus (10, 11). The most common origin site of these organisms, both in the vagina and intestinal tract, is the anterior nares (1, 12).

Various lactobacilli and related bacteria are the dominant microbiome organisms in both the vagina and intestinal tract (13–15). For example, women are typically colonized with 5 × 10^7^ vaginal lactobacilli per tampon, and these organisms persist throughout the menstrual cycle (13), even during menstruation, when pathogens such as TSS S. aureus may grow to even higher numbers, reaching even 10^11^ per tampon (16).

In our prior studies, we have observed that some women have only lactobacilli present in the vagina (17). These women do not appear able to be colonized with potential pathogens on this mucosal surface. These observations suggest that it may be possible to colonize women on mucosal surfaces with probiotic lactobacilli to reduce the presence of TSS S. aureus. Two such probiotic lactobacilli are Lacticaseibacillus rhamnosus strain HN001 and Lactobacillus acidophilus strain LA-14. Furthermore, the combination of these probiotic strains has shown beneficial effects on vaginal health, particularly in women with dysbiotic vaginal microbiota, in randomized placebo-controlled clinical trials (18, 19). However, the effects of these probiotics on TSS-associated S. aureus or TSST-1 production are unclear.

This study was undertaken to determine the *in vitro* effects of both L. rhamnosus and Lactobacillus acidophilus, both separately and when combined, on TSS S. aureus, its production of TSST-1, and the ability of TSST-1 to induce pro-inflammatory chemokine production by human vaginal epithelial cells (HVECs). L. rhamnosus HN001 prevented TSST-1 production, partially through acid production, but did not inhibit growth of TSS S. aureus. In contrast, L. acidophilus LA-14 both killed TSS S. aureus and simultaneously prevented TSST-1 production, partially through acid and H_2_O_2_ production. When it was incubated together with TSS S. aureus, the effect of L. acidophilus dominated. L. acidophilus did not kill L. rhamnosus. Finally, neither lactobacillus alone induced interleukin-8 (IL-8) chemokine production by HVECs, or induced only low-level production, but both lactobacilli inhibited IL-8 production by HVECs, as induced by TSST-1.

## RESULTS

### Growth of L. rhamnosus HN001, L. acidophilus LA-14, and S. aureus.

In our first set of studies, the stationary phase of all three organisms was determined after growth in Todd Hewitt (TH) medium with shaking (150 rpm) at 37°C. The inoculum size was estimated to be 10^7^ CFU/mL and then verified by serial dilution plate counts. After 48 h, L. rhamnosus had grown to approximately 10^9^ CFU/mL. The colonies, as grown on chocolate agar, were approximately 2 mm in diameter after 48 h incubation in a 5% CO_2_ incubator. In contrast, L. acidophilus grew to only 2 × 10^8^ CFU/mL after the 48-h growth period. When plated on chocolate agar, the L. acidophilus colonies remained as pinpoints, even after incubation for up to 1 week in the presence of 5% CO_2_. These stationary phases agreed with those provided by the commercial source of the lactobacilli.

The stationary phase of USA200 (CC30) TSS S. aureus MN8 (20) was determined to be approximately 7 × 10^9^ CFU/mL after 24 h culture in Todd Hewitt broth at 37°C with 150-rpm shaking. The starting inoculum was 1.2 × 10^7^ CFU/mL, the approximate average CFU/mL found in the vagina during menstruation. The colonies grew on sheep blood agar plates as relatively non-hemolytic colonies about 2 mm in size, as expected for this organism. Three additional strains of USA200 (CC30) S. aureus strains were cultured similarly to MN8 and their stationary phases were determined. CDC587 (7) and Harrisburg (7, 21), from menstrual TSS cases, and a clinical isolate from enterocolitis/TSS (10, 11) had stationary phases of 5.3 × 10^9^, 7.8 × 10^9^, and 9.8 × 10^9^ CFU/mL, respectively. Their appearances on blood agar plates were similar to that of strain MN8.

### L. acidophilus LA-14 and L. rhamnosus HN001 growth kinetics.

**FIG 1:**
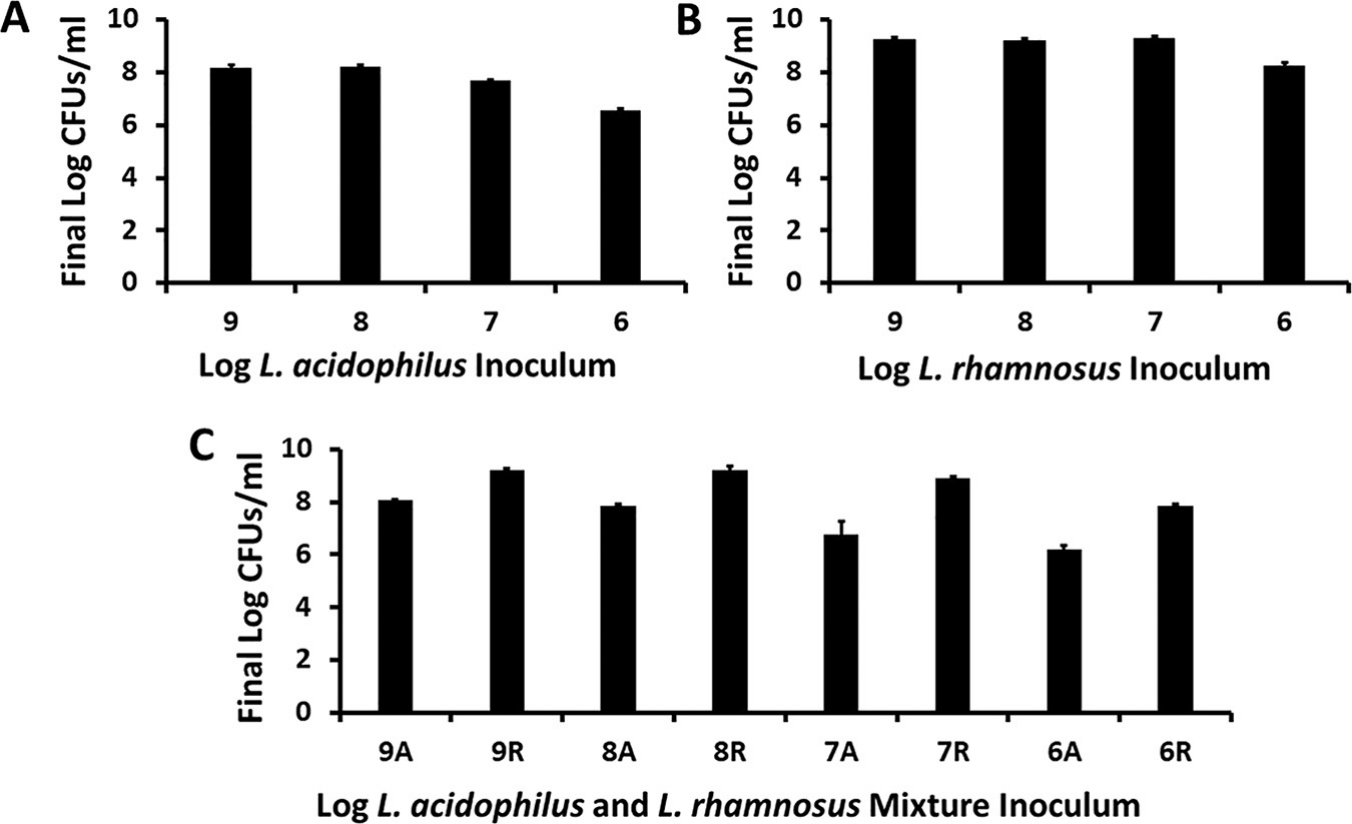
Growth kinetics of (A) L. acidophilus and (B) L. rhamnosus alone or (C) as mixtures after 48 h of incubation with 150-rpm shaking in a standard 37°C incubator. Bars indicate means ± standard deviation. Numbers on the abscissas indicate log_10_ of the starting inocula for L. acidophilus and L. rhamnosus alone or when the two organisms were incubated as a mixture (A, L. acidophilus and R, L. rhamnosus). Log_10_ of 1.0 equals the lower limit of detection of lactobacilli.

Initially, the growth of the two lactobacillus strains was determined after 24 h of incubation at 37°C with shaking at 150 rpm, as incubated separately and when mixed at various starting inocula (Fig. 1A to C). The 24-h time point was chosen as the end of experiment because all four S. aureus strains, used in subsequent experiments, achieved their stationary phases before 24 h. L. acidophilus at 10^9^ CFU/mL dropped to 10^8^ CFU/mL, its usual stationary phase (Fig. 1A). L. acidophilus at 10^8^ CFU/mL remained at 10^8^ CFU/mL. L. acidophilus at starting inocula of 10^7^ and 10^6^ CFU did not reach the expected stationary phase by 24 h.

In contrast, L. rhamnosus at inocula of 10^9^, 10^8^, and 10^7^ CFU/mL all achieved the expected stationary phase (Fig. 1B). Only the L. rhamnosus 10^6^-CFU/mL inoculum did not reach the stationary phase by 24 h of incubation.

When equivalent CFU/mL of both organisms were mixed together and incubated for 24 h, the growth patterns for each remained the same as when they were cultured separately (Fig. 1C). This indicated that neither organism was interfering with the growth of the other.

### Effect of *L. acidophilus* LA-14 and *L. rhamnosus* HN001 on growth of *S. aureus*.

**FIG 2:**
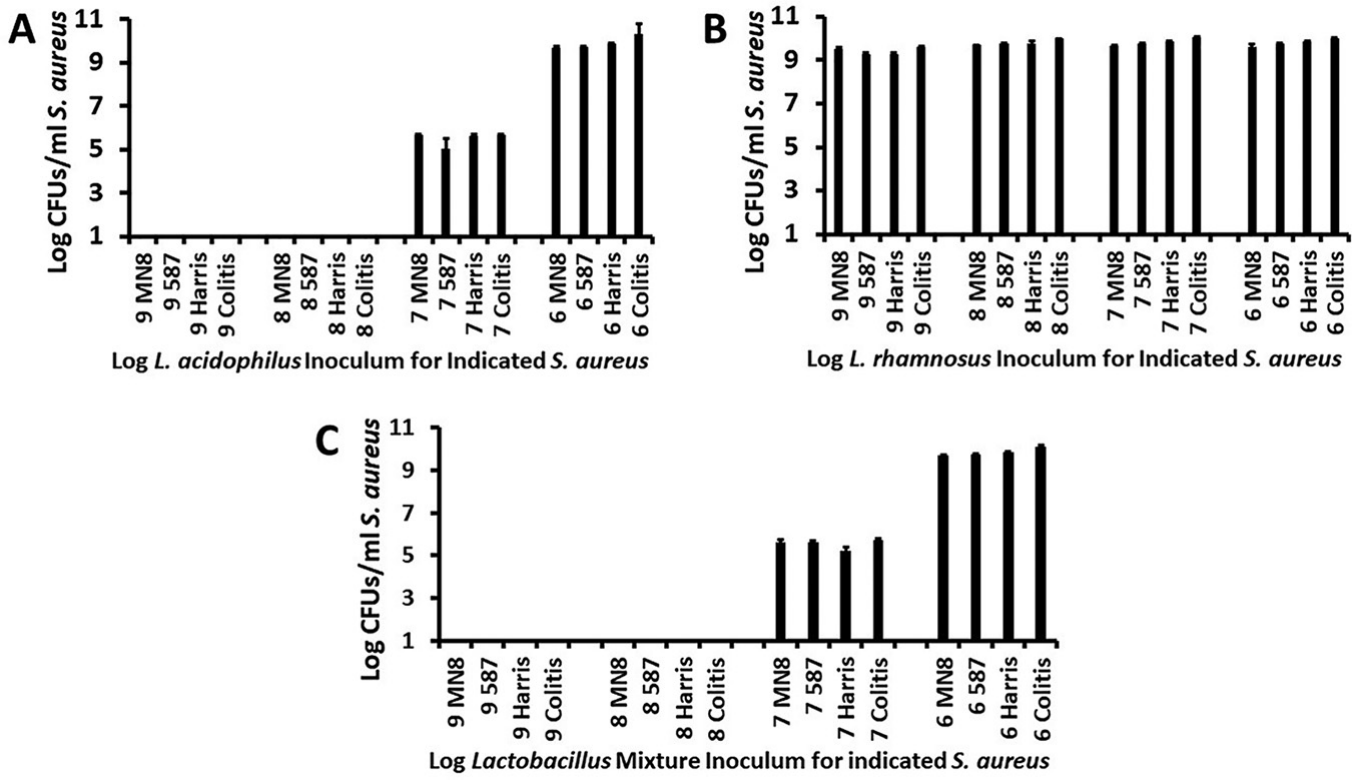
Effect of (A) L. acidophilus + S. aureus strains, (B) L. rhamnosus + S. aureus strains, or (C) an equal mixture of the two lactobacilli + S. aureus strains on the 24-h growth of various S. aureus strains. S. aureus strains included USA200 (CC30) MN8, CDC587 (587), Harrisburg (Harris), and an enterocolitis isolate (Colitis) after 24 h of incubation with shaking at 150 rpm in a standard 37°C shaker. Log_10_ of 1.0 equals the lower limit of detection of S. aureus. Numbers on the abscissas indicate the log_10_ of the starting inocula for L. acidophilus and L. rhamnosus alone or when the two organisms were incubated as a mixture.

Next, the same starting inocula of L. acidophilus and L. rhamnosus and a mixture of both at the same inoculum density were mixed together with approximately 10^7^/mL of each of the four S. aureus strains individually and cultured for 24 h in Todd Hewitt broth. Lactobacilli were inoculated 2 h prior to the addition of S. aureus to give the lactobacilli a growth head-start. This makes sense because lactobacilli are constantly present on mucosal surfaces (14), such as the vaginal mucosa (5 × 10^7^/mL) and the large intestine, whereas S. aureus are typically present intermittently (12, 16, 22). There are occasional persons who are permanently colonized by S. aureus, but these are a minority. At the 24-h incubation time point at 37°C with 150-rpm shaking, the CFU/mL of all S. aureus organisms in the mixtures was determined (Fig. 2A to C).

L. acidophilus was highly antimicrobial against all S. aureus strains (Fig. 2A). L. acidophilus starting inocula of 10^9^ and 10^8^ CFU/mL were bactericidal against S. aureus, as evidenced by a >3-log reduction in S. aureus CFU/mL after 24 h of incubation. The L. acidophilus starting inoculum of 10^7^ CFU/mL was bacteriostatic against S. aureus, as indicated by the lack of growth, but the viability of S. aureus was retained over the 24-h test period (Fig. 2A). L. acidophilus at 10^6^ CFU/mL did not negatively affect the growth of S. aureus (Fig. 2A). S. aureus strains had no effects on the growth of L. acidophilus at any *Lactobacillus* starting inoculum; these data were similar to those shown in Fig. 1.

L. rhamnosus alone at any starting inoculum had no effect on the growth of any of the four S. aureus strains (Fig. 2B). Similarly, S. aureus did not interfere with the growth of L. rhamnosus at any *Lactobacillus* starting inoculum size; L. rhamnosus CFU/mL were similar to those shown in Fig. 1.

When equivalent numbers of L. acidophilus and L. rhamnosus were incubated for 24 h with S. aureus strains, S. aureus numbers reflected the antimicrobial activity of incubation with L. acidophilus alone (Fig. 2C). Thus, when L. acidophilus was present at 10^9^ and 10^8^ CFU/mL with equivalent numbers of L. rhamnosus, the mixture was bactericidal against S. aureus, but the two lactobacilli exhibited no inhibitory effect on each other. When L. acidophilus was present in the mixture at 10^7^ CFU/mL, the mixture of lactobacilli was bacteriostatic against S. aureus (Fig. 2C). Finally, when the mixture contained 10^6^/mL of each lactobacillus, the growth of S. aureus was not affected (Fig. 2C). Similarly, S. aureus strains did not interfere with the growth of the mixture of L. acidophilus and L. rhamnosus at any *Lactobacillus* starting inoculum size; lactobacilli CFU/mL were similar to those shown in Fig. 1.

### Inhibition of TSST-1 production by lactobacilli.

**FIG 3:**
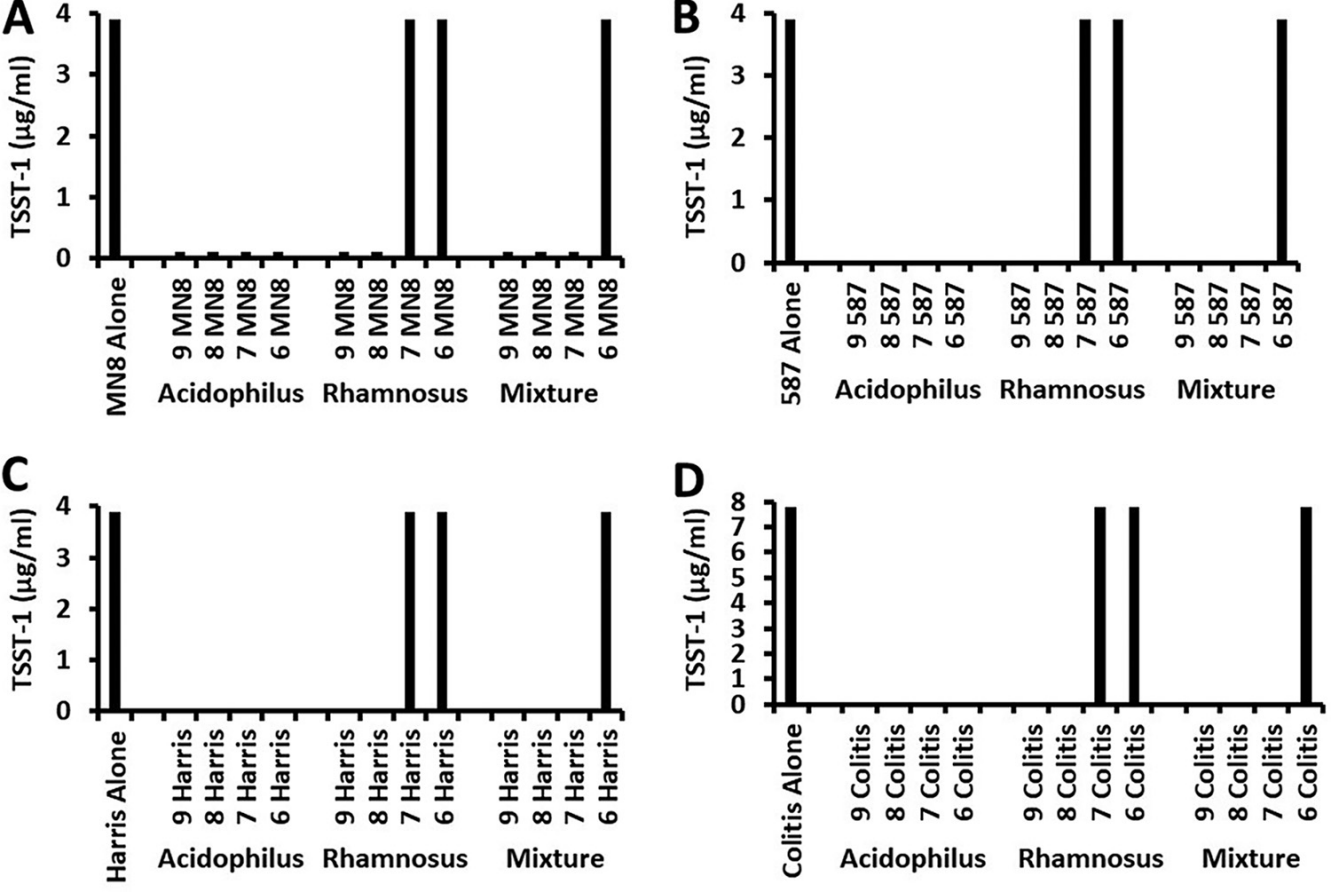
Effect of lactobacilli on production of TSST-1 by S. aureus as tested by quantitative double immunodiffusion. TSST-1 production by the four S. aureus strains in the absence of lactobacilli ([A] MN8 Alone, [B] CDC587 Alone [587 Alone], [C] Harrisburg Alone [Harris Alone], and [D] Colitis Alone); in the presence of L. acidophilus (log CFU/mL = 9 to 6); in the presence of L. rhamnosus (log CFU/mL = 9 to 6); or in the presence of equal mixtures of L. acidophilus and L. rhamnosus (Mixture; log CFU/mL = 9 to 6). The lower limit of detection of TSST-1 by this assay was 0.6 μg/mL.

We also tested the ability of the two lactobacilli, inoculated prior to the S. aureus strains, to inhibit TSST-1 production using a quantitative double immunodiffusion method (20) (Fig. 3A to D). At all 4 concentrations of L. acidophilus alone cultured with any S. aureus strain, no TSST-1 was detected (the lower limit of detection was 0.6 μg/mL original culture fluid). Even the lowest concentrations of L. acidophilus LA-14, a concentration that was not bactericidal for S. aureus, this lactobacillus inhibited TSST-1 production below the 0.6-μg/mL lower limit. In contrast, S. aureus strains alone as controls produced approximately 4 to 8 μg/mL TSST-1. Since no standard deviation was observed using this method, the differences in TSST-1 levels from cultures with lactobacilli present compared to those with S. aureus alone were highly significant.

Even though L. rhamnosus HN001 at all concentrations had no effect on S. aureus growth, at the two highest concentrations (10^9^ CFU/mL and 10^8^ CFU/mL), this organism completely inhibited the production of TSST-1 (Fig. 3A to D). At the two lower CFU/mL of L. rhamnosus, the lactobacilli had no inhibitory effect on TSST-1 production.

When the mixture of the lactobacilli was incubated with S. aureus, the three highest concentrations of lactobacilli completely prevented TSST-1 production (Fig. 3A to D). However, the lowest concentration of the mixture of lactobacilli (starting inoculum of each was approximately 10^6^ CFU/mL) had no inhibitory effect on TSST-1 production.

### Effect of lactobacilli on *S. aureus* MN8 and the enterocolitis isolate growth, and on TSST-1 production when *S. aureus* was inoculated 1 h before lactobacilli.

**FIG 4:**
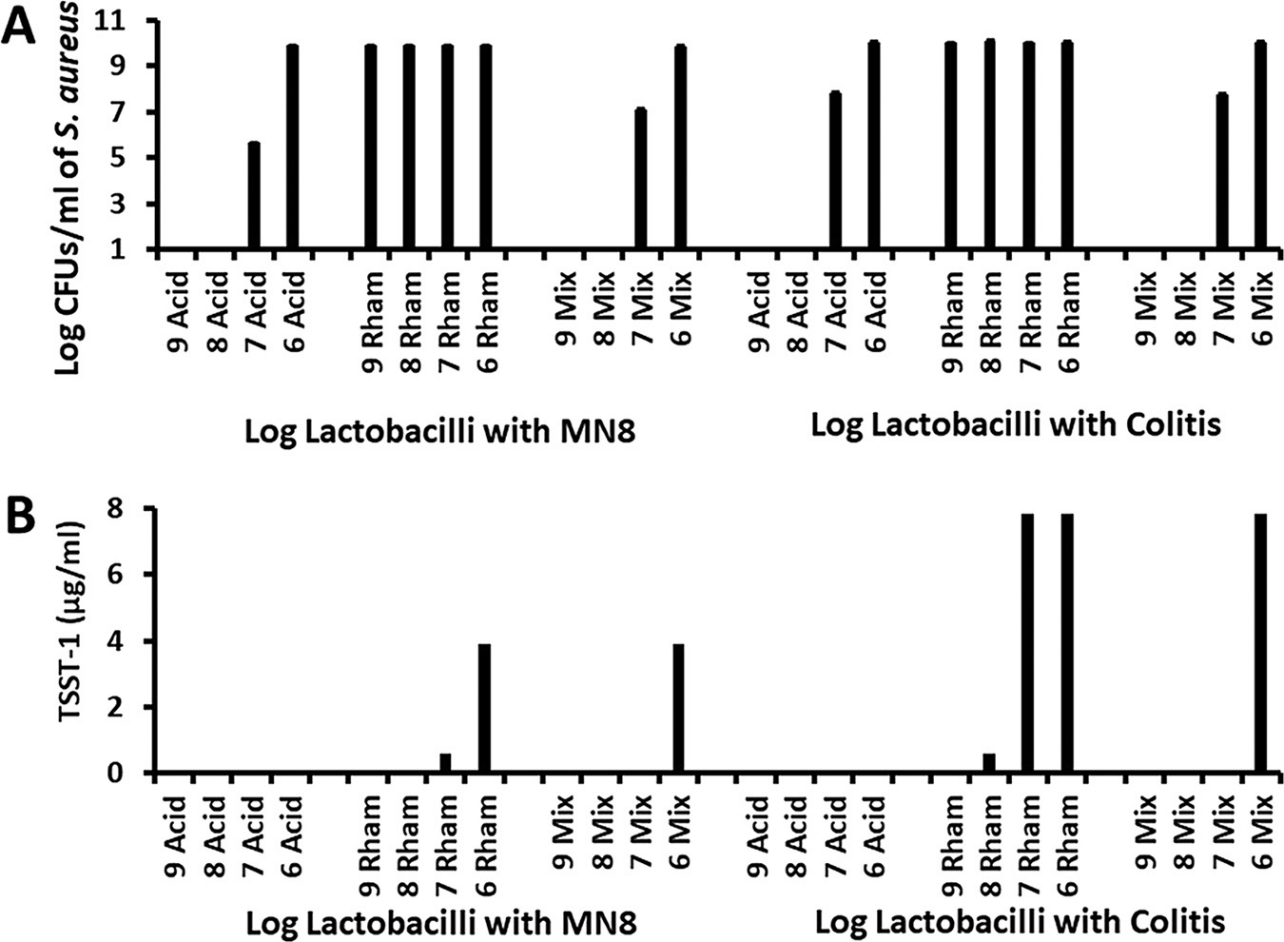
Effect of lactobacilli on (A) the growth of TSS S. aureus and (B) TSST-1 production when S. aureus strains were cultured 1 h prior to addition of lactobacilli. S. aureus strains tested were MN8 and the strain from enterocolitis (Colitis). MN8 inoculum was 1.2 × 10^7^ CFU/mL and Colitis inoculum was 1.4 × 10^7^ CFU/mL. Lactobacilli were L. acidophilus (Acid), L. rhamnosus (Rham), or equal mixtures of both lactobacilli (Mix). Bars indicate standard deviations. There were no standard deviations in the measurement of TSST-1. Numbers on the abscissas are the log_10_ numbers of lactobacilli present in the cultures.

Our experience is that nearly all women of menstrual age, with only one exception who was colonized with a pure culture of Enterococcus faecalis, have approximately 5 × 10^7^ CFU of lactobacilli present in the vagina, both during menstruation and at other times. Similarly, lactobacilli are present in the human large intestine. However, not all persons would be expected to be colonized on these mucosal surfaces with the two specific lactobacilli examined in our current study. Thus, we evaluated the effects of the two lactobacilli on vaginal isolate MN8 and an enterocolitis isolate when S. aureus was inoculated into Todd Hewitt broth prior to the addition of lactobacilli (Fig. 4A and B). We used the same starting inocula for both S. aureus strains, as in prior experiments. However, we could only give the staphylococci a 1-h growth head-start for the following reason: the two S. aureus strains double every 20 min, thus, if we gave the strains a 2-h head-start, the organisms would have already been producing TSST-1 (production begins at approximately 5 × 10^8^ CFU/mL) prior to the addition of lactobacilli.

The lactobacilli, when added to the cultures 1 h after S. aureus, had the same effect on both S. aureus strains (Fig. 4A) as when they were added 2 h prior to S. aureus. L. acidophilus alone inhibited the growth of both S. aureus strains at the three highest concentrations tested (Fig. 4A). Additionally, production of TSST-1 was inhibited at all four lactobacillus concentrations (Fig. 4B).

At all four concentrations, L. rhamnosus did not inhibit the growth of either S. aureus strain (Fig. 4A), but it did inhibit TSST-1 production at the two higher concentrations (Fig. 4B).

Finally, the mixture of both lactobacilli completely inhibited or delayed growth at the three highest lactobacillus concentrations (Fig. 4A) and completely inhibited TSST-1 production at the three highest concentrations (Fig. 4B).

Collectively, the data shown in Fig. 1 to 4 suggest that the L. acidophilus strain LA-14 strongly inhibited both TSS S. aureus growth and TSST-1 production. In contrast, the L. rhamnosus strain HN001 did not inhibit TSS S. aureus growth but was able to inhibit TSST-1 production. A mixture of the two lactobacilli had intermediate effects on TSS S. aureus growth and TSST-1 production, with the highest concentrations being inhibitory. This was the case whether lactobacilli were added to the medium before or after the addition of TSS S. aureus.

### Potential mechanism of interference with *S. aureus* growth and TSST-1 production.

Studies were initiated with L. acidophilus LA-14 and L. rhamnosus HN001 to determine the possible mechanisms of their interference with S. aureus MN8 growth using a representative USA200 (CC30) strain (for L. acidophilus only) and, additionally, their effects on TSST-1 production due to MN8 exposure to each lactobacillus individually.

Previously, we have shown that TSST-1 production is inhibited by the growth of S. aureus in conditions of pH 6.5 or lower (23). It was hypothesized that this could partly account for the effects of L. rhamnosus observed on S. aureus MN8 production of TSST-1. The pHs of the cultures of L. rhamnosus alone (10^9^ to 10^6^ CFU/mL), S. aureus MN8 alone, and a mixture of L. rhamnosus and S. aureus MN8 were determined after 24 h of culture. L. rhamnosus (10^9^ and 10^8^ CFU/mL) reduced the pH of the Todd Hewitt broth to approximately 6.0 after 24 h, but 10^7^ and 10^6^ CFU/mL did not reduce the pH below the starting pH of 7.5. The two higher L. rhamnosus concentrations inhibited TSST-1 production while not affecting S. aureus growth. The final pH of the S. aureus MN8 culture alone was 6.4. The cultures of L. rhamnosus (10^9^ and 10^8^ CFU/mL) + S. aureus MN8 had final pHs of approximately 6.0. The cultures of L. rhamnosus (10^7^ and 10^6^ CFU/mL) + S. aureus MN8 had final pHs of approximately 6.5.

**FIG 5:**
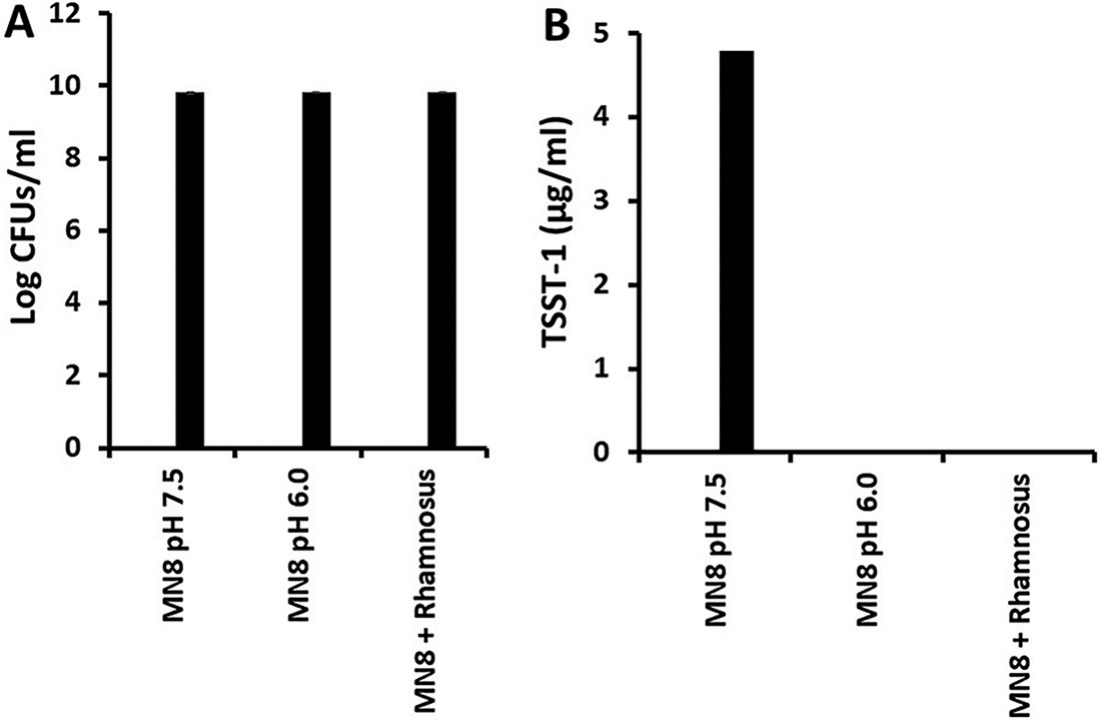
Effect of pH on (A) growth of S. aureus MN8 and (B) production of TSST-1. MN8 (1.2 × 10^7^/mL starting inoculum) when cultured for 24 h at the indicated pH or in the presence of 10^9^/mL L. rhamnosus at 37°C with shaking (150 rpm). Data are presented as means of log CFU/mL ± standard deviation and TSST-1 μg/mL. The coculture of L. rhamnosus + S. aureus MN8 had a final pH of 5.9.

Thus, we examined the growth of S. aureus MN8 alone after 24 h with pHs maintained at 7.5 and 6.0, and S. aureus with an additional culture of L. rhamnosus (10^9^ CFU/mL) + MN8 (starting inoculum 1.2 × 10^7^ CFU/mL) (Fig. 5). S. aureus grew to the expected final concentrations of approximately 7.5 × 10^9^ CFU/mL, whether at pH of 7.5 or 6.0, or in the presence of L. rhamnosus (final pH determined to be 5.9). In contrast, TSST-1 was only produced in the S. aureus cultures maintained at pH 7.5.

**FIG 6:**
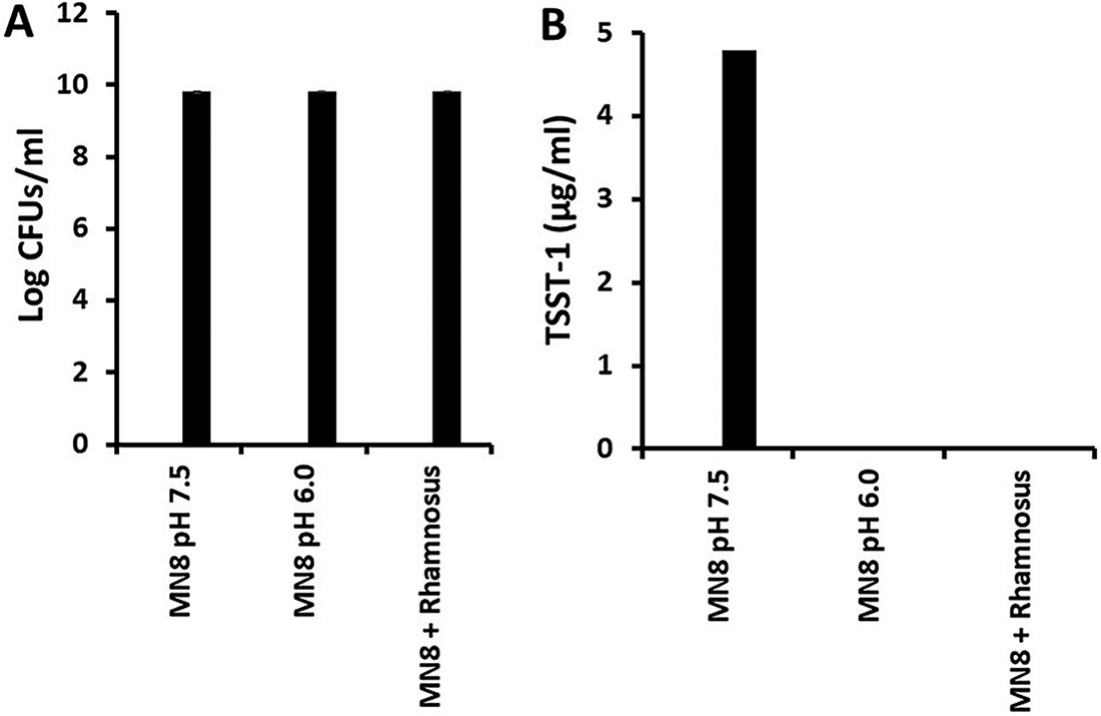
Effect of L. rhamnosus on (A) growth and (B) TSST-1 production by S. aureus MN8 and MN8Δ*srr.* L. rhamnosus (10^9^ CFU/mL) was inoculated 2 h ahead of S. aureus MN8 (starting inoculum 1.1 × 10^7^/mL) and MN8Δ*srr* (starting inoculum 1.2 × 10^7^/mL). All cultures were incubated at 37°C with shaking (100 rpm) for 24 h. At the 24-h time point, pH, S. aureus CFU/mL, and TSST-1 μg/mL were determined.

A global regulator of TSST-1 production is the SrrA/B two-component system (24). It is believed this two-component system indirectly senses oxygen through redox potential in the membrane. It was hypothesized that the pH effect of L. rhamnosus on TSST-1 production may partly depend on SrrA/B. This was evaluated through the growth of S. aureus MN8 (1.1 × 10^7^ CFU/mL starting inoculum) and an SrrA/B knockout in MN8 (MN8Δ*srr*; 1.2 × 10^7^ CFU/mL starting inoculum) in the presence of L. rhamnosus for 24 h at 37°C with 150-rpm shaking. L. rhamnosus (1.0× 10^9^ CFU/mL starting inoculum) was inoculated 2 h prior to S. aureus MN8. An additional control (for pH), consisting of L. rhamnosus alone, was treated similarly except for the absence of S. aureus. The final pH of all treatment groups ranged from 5.9 to 6.1. S. aureus MN8 and MN8Δ*srr* both grew to approximately 6.6 × 10^9^ CFU/mL (Fig. 6A). However, no detectable TSST-1 was measured in the cultures containing MN8 + L. rhamnosus (Fig. 6B). In contrast, MN8Δ*srr* + L. rhamnosus contained 4.8 μg/mL of TSST-1 (Fig. 6B). These data suggest the TSST-1-inhibitory effect of L. rhamnosus depends directly or indirectly on SrrA/B.

Next, the pH of the Todd Hewitt growth medium was measured after incubating L. acidophilus for 24 h. The highest concentrations of L. acidophilus (10^9^ CFU/mL) reduced the pH of the medium to approximately 5.5. This pH is bacteriostatic for S. aureus MN8. However, this same L. acidophilus concentration was bactericidal for S. aureus MN8 (Fig. 2). This suggested that pH reduction alone may account for only part of the inhibition of S. aureus MN8 growth. It could not explain the complete killing effect.

Another major end product of L. acidophilus metabolism, in addition to acid production, is the production of H_2_O_2_. Thus, the pH and H_2_O_2_ levels of Todd Hewitt medium were determined after 24 h culture in triplicate of L. acidophilus (10^9^ CFU/mL). The final pH was 5.6. The concentration of H_2_O_2_ averaged 0.01% (vol/vol).

Subsequently, S. aureus MN8 (1.1 × 10^7^ CFU/mL starting inoculum) was cultured alone in Todd Hewitt medium maintained at pH 5.5 or in the same medium maintained at pH 5.5 + the addition of 0.01% H_2_O_2_. S. aureus MN8 in the cultures maintained at pH 5.5 without 0.01% H_2_O_2_ were not killed, but they did not grow (final CFU = 1.5× 10^7^/mL). However, in the presence of both acidic pH and 0.01% H_2_O_2_, S. aureus MN8 were killed by more than 3 logs (final CFU/mL = 3.1× 10^2^ CFU/mL; *P* < 0.0001 for differences of means by Student’s *t* test). These data indicate that both the acidic pH and H_2_O_2_ contributed to the killing of S. aureus MN8. However, additional factors, for example, tetramic acids and bacteriocins, must be contributing. As expected, no TSST-1 was detected in either set of cultures because TSST-1 is only produced during the typical post-exponential-growth phase beginning at approximately 5× 10^8^ CFU/mL.

### Effects of lactobacilli on IL-8 production by HVECs alone or in combination with TSS *S. aureus*.

**FIG 7:**
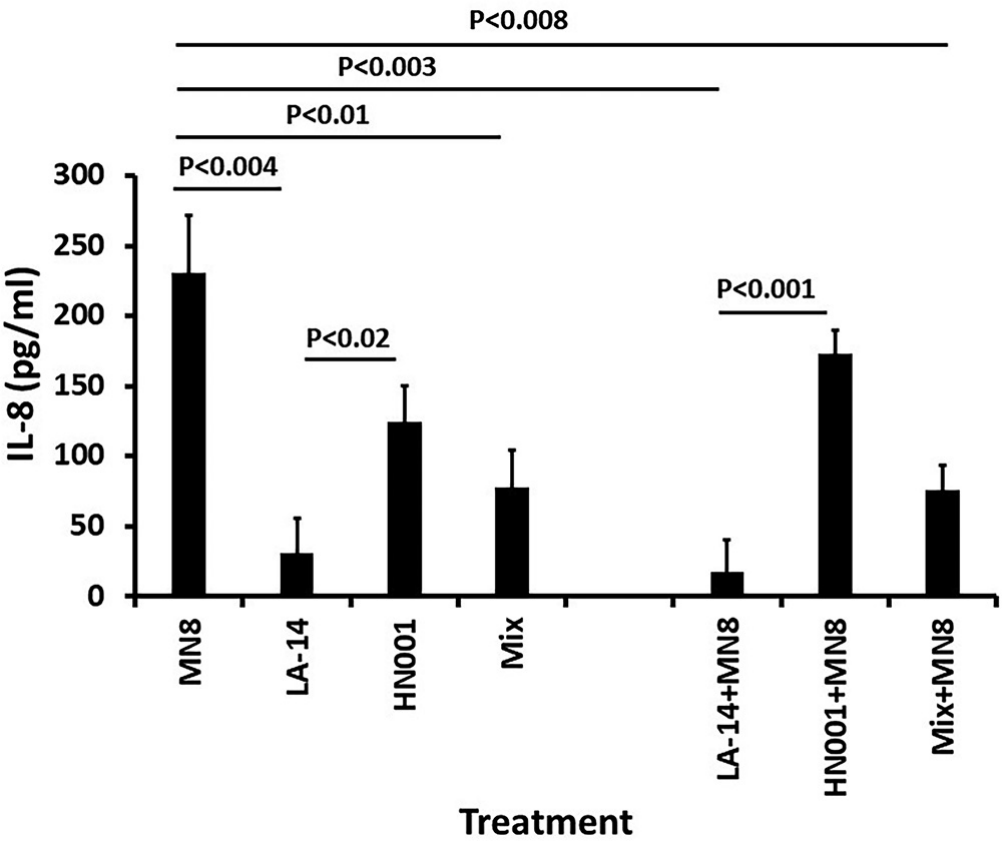
Effect of lactobacilli on IL-8 production by HVECs alone or in combination with TSS S. aureus strain MN8. Bars indicate means ± standard deviation from triplicate 96-well confluent cultures of HVECs in keratinocyte serum-free medium. Differences in means (*P* values) were determined by Student’s *t* test. Treatments included S. aureus MN8 alone, L. acidophilus (LA-14) alone, L. rhamnosus (HN001) alone, and a mixture (Mix) of both lactobacilli; and MN8 with L. acidophilus (LA-14+MN8), L. rhamnosus (HN001+MN8), or a mixture of both lactobacilli (Mix+MN8). Incubation time was 6 h at 37°C in the presence of 5% CO_2._

We tested the effect of the two lactobacilli alone, as a mixture, and in combination with S. aureus MN8 for their ability to induce the production of the chemokine IL-8 (Fig. 7). None of the organisms, as added to the HVECs, killed the HVECs during the 6-h incubation period. Thus, the lack of responses was not the result of HVEC death.

Neither lactobacillus alone (10^9^ CFU/mL) nor a mix of both at 10^9^ CFU/mL induced pro-inflammatory IL-8 production to the extent of S. aureus MN8 alone (7.5× 10^8^ CFU/mL), although induction by L. rhamnosus alone barely reached statistical significance (*P* < 0.04) compared to MN8. L. acidophilus alone induced significantly less IL-8 production than L. rhamnosus. When tested alone and when mixed with L. rhamnosus, L. acidophilus strongly reduced chemokine production by S. aureus MN8. L. rhamnosus alone reduced IL-8 production compared to MN8, but the difference was not significant. L. acidophilus alone was better able to reduce IL-8 production in the presence of MN8 than L. rhamnosus in the presence of MN8.

## DISCUSSION

Staphylococcal TSS is an acute-onset, multi-organ illness that occurs primarily due to TSST-1-producing S. aureus on mucosal surfaces (2, 7, 8). There are many subsets of mucosa-associated TSS, including menstrual (7, 8), post-influenza (25), and enterocolitis-associated (10, 11). Menstrual TSS is 100% TSST-1-associated and is more likely to occur in 12- to 15-year-old preteens and teenagers, but the disease can occur at any age in association with menstruation (7, 8).

It has been shown that as many as 20% of women worldwide do not produce antibodies against TSST-1 that would provide systemic immunity against the massive CD4 T lymphocyte and macrophage activation required for TSS symptoms (2, 9, 26–28). It has also been shown that the initial human cell type affected by TSST-1 that sets menstrual TSS production in motion is the vaginal epithelial cell (29–31). These cells produce chemokines which facilitate the penetration of TSST-1 across the mucosal barrier through their interaction with the immune co-stimulatory molecule CD40 (30).

The environmental factors required for TSST-1 production are well-known (23, 32). These include temperatures near 37°C, or higher temperatures, as seen in the presence of fevers. Superantigens such as TSST-1 are among the most potent pyrogens known (2). Another factor required for TSST-1 production is a pH greater than 6.5 and less than 9.0. We have shown that the vaginal pH in young women rises to around 7.2 within a day or two of menstruation and remains there during the entire menstrual period, dropping a day or two after menstruation ends. Protein is required for TSST-1 production, although the blood present in tampons appears to interfere with its production (32). It is thus hypothesized that protein in the vaginal secretions in tampon regions without significant blood levels are where the most TSST-1 is produced (32). Finally, multiple studies have suggested that tampons are associated with menstrual TSS because they oxygenate the vaginal environment (23, 33). This does not appear to be oxygen inserted along with the tampon, but rather oxygen that is trapped within the fibers (34). The “oxygen” theory for tampon association with menstrual TSS also explains the increased risk associated with increased absorbency. Additionally, it has been shown that oxygen is required for the production of secreted virulence factors, including TSST-1 (23, 35).

The regulation of TSST-1 production by oxygen appears to be related to the de-repression of the SrrA/B two-component regulator system (24, 36). In the absence of oxygen, SrrA/B repression prevents TSST-1 production. However, in the presence of oxygen, SrrA/B no longer represses expression. Mutants of SrrA/B have been isolated (37) which produce TSST-1 even in the absence of oxygen. It is unlikely that SrrA/B directly senses oxygen in the environment. Instead, the system more likely senses the redox potential within the plasma membrane (38). This would be consistent with the observations that menaquinone analogs inhibit TSST-1 production (38). These data would also be consistent with the pH effect observed in the current study.

In the current study, we show that probiotic lactobacilli interfere with TSS S. aureus growth, as in the case of L. acidophilus. For this organism, both acid production and H_2_O_2_ explain much of the growth-inhibitory effect. However, it is likely that other factors, possibly the tetramic acids produced by some lactobacilli, facilitate the complete inhibition of growth (17, 39, 40). Tetramic acids such as reutericyclin can be produced by lactobacilli and some enterococci (17, 39, 40). Reutericyclin is broadly antimicrobial unless an immunity gene is present in the target organism. Thus far, reutericyclin appears generally restricted to lactobacilli and at least one E. faecalis strain on mucosal surfaces.

Prior studies have shown that vaginal conditions, for example, those that cause bacterial vaginosis and candidiasis, can be reduced, and recurrences controlled by treatment with the lactobacilli used in this study in combination with lactobacilli and lactoferrin (19, 41, 42). From genomic analyses, it is known that L. acidophilus LA-14 can produce two antimicrobial bacteriocins, enterocyclin A and helveticin J. It is possible that at least some effects of L. acidophilus LA-14 on TSS S. aureus are due to these proteins.

Additionally, it has been suggested that vaginal lactobacilli may interfere with the production of TSST-1 (43, 44).The effect, at least as caused by L. reuteri, may be partly due to the action of cyclic dipeptides on the global regulatory and two-component system referred to as the accessory gene regulator. As mentioned above, tetramic acids may contribute to the ability of L. reuteri to interfere with TSS S. aureus; the name of reutericyclin (a tetramic acid) is derived from its production by L. reuteri.

In the case of L. rhamnosus, no growth-inhibitory effect was observed with respect to TSS S. aureus. However, acid production by this organism accounted for much of its ability to inhibit TSST-1 production.

Importantly, neither L. acidophilus nor L. rhamnosus interfered with each other’s growth. Thus, they make a suitable probiotic combination. In addition, the combination of these probiotics has been investigated in several randomized placebo-controlled clinical trials in women for their potential vaginal benefits, and no safety concerns have been raised in the context of these studies (18, 19, 41, 45).

Another aspect for consideration in this study is where these probiotics may be used to improve health. Clearly, they can be taken orally to help alter the gut microbiome, possibly to help prevent enterocolitis associated with TSS S. aureus (10, 11). It is well known that the majority of enterocolitis is associated with Clostridioides difficile (46, 47). However, prior to the identification of C. difficile causing the majority of cases, staphylococcal superantigens were considered important causative factors (10, 11). While the role in enterocolitis of S. aureus superantigens, including TSST-1, has been largely forgotten, these factors clearly continue to have an association (10, 11). It is possible that the combination of the two lactobacilli could help reduce the incidence of such staphylococcal-associated enteric disease.

A group of women has been identified who possess only lactobacilli or E. faecalis in the vagina (17). Potential pathogens, including S. aureus, are not isolated vaginally from these women. Thus, it would be expected that these women would not be at risk for menstrual TSS. It is known that during menstruation, many potential pathogens grow to very large numbers. This includes S. aureus, group B streptococci, and Escherichia coli (48). This is thought to occur because normal microbiome lactobacilli can no longer maintain a vaginal pH of 4.0 to 4.5 as they do at times other than menstruation. However, this small group of women with broadly antimicrobial lactobacilli do not have the risk of these potential pathogens growing to large numbers. Thus, it is possible that attempts to recolonize women with a combination of probiotic lactobacilli may reduce the incidence of not only menstrual TSS, but also other infections originating from the vaginal environment.

Importantly also, we have shown that vaginal pathogens, such as S. aureus and HIV, induce pro-inflammatory cytokines, including IL-8, to facilitate the penetration of TSST-1 and simian immunodeficiency virus across the multi-layered vaginal mucosa (49–51). We have referred to this enhanced penetration as “outside signaling” where harmful host inflammation enhances disease production (31). With this in mind, it is important to consider that the lactobacilli used in the current study induce only minimal pro-inflammatory IL-8 production by HVECs by themselves; at the same time, these two lactobacilli, particularly L. acidophilus alone or a mixture of both lactobacilli, significantly reduce the ability of S. aureus MN8 to induce IL-8 production.

Overall, these two probiotic lactobacilli have two potentially important positive effects that hopefully can be translated into effects in humans. The first of these is reduction of TSST-1 production, and the second is inhibition of potentially harmful pro-inflammatory cytokines.

## MATERIALS AND METHODS

### Bacteria.

S. aureus strains MN8, CDC587, and Harrisburg are menstrual TSS isolates from the early 1980s (7, 20, 21). A clinical isolate from enterocolitis was obtained from a patient with no C. difficile isolated but instead TSST-1 producing S. aureus (10, 11). S. aureus MN8 with the gene for the two-component system SrrA/B knocked out has been previously described (38). All organisms were of low passage number and stored at −80°C in the Schlievert laboratory. For experimentation, the staphylococci were cultured in TH broth at 37°C with shaking (150 rpm) in a standard shaker. Serial dilution plate counts on blood agar were used to quantify S. aureus. This laboratory has used these organisms extensively, so the stationary phases could be estimated. However, the actual stationary phases of all organisms were determined by serial dilution plates counts after 24 h of incubation under the conditions described above, with starting inocula estimated to be approximately 10^7^/mL.

L. acidophilus LA-14 and L. rhamnosus HN001 were generously provided by USPL Nutritionals, LLC as lyophilized samples. These bacteria were likewise cultured in TH broth at 37°C with shaking (150 rpm) in a standard shaker. Serial dilution plate counts on chocolate agar were used to quantify these microbes; the chocolate agar plates containing lactobacilli were cultured in a 5% CO_2_ incubator for 48 h. The lactobacilli provided were quantified by the manufacturer prior to lyophilization. However, the actual numbers (CFU/mL) were determined by serial dilution plate counts on chocolate agar after growth in TH broth with shaking at 37°C for 48 h.

### Chemicals.

Pluronic L92 was provided by the BASF Corporation. H_2_O_2_ was purchased from Sigma-Aldrich. The colorimetric assay for H_2_O_2_ (Pierce Quantitative Peroxide Assay) was purchased from Thermo Fisher Scientific (Rockford, IL), and assays were performed exactly as provided by the manufacturer.

In some experiments, the pHs of the media were maintained at pH 7.5 or 6.0 by continuous addition of phosphate (0.1 M NaPO_4_ [pH 8.0]) or acetate (0.1 M sodium acetate [pH 4.5]). The pHs of the original TH broths were adjusted to the desired pH before maintenance of pH by buffer addition. The pHs of the media were measured every 6 h, including the final pH, to ensure that pH was maintained.

### TSST-1 measurement.

A quantitative double immunodiffusion assay was used to assess TSST-1 levels (20, 23). The first was a semiquantitative double immunodiffusion assay, with a lower limit of detection of 6.0 μg/mL for TSST-1. To increase the sensitivity of this assay, culture fluids (after microbial growth) were treated with 80% final concentration of ethanol to precipitate proteins of >10,000 molecular weight. It was previously shown that that this method precipitates 100% of measurable TSST-1 (32). The precipitated proteins were restored with pyrogen-free distilled water to 1/10 of the original volume. This increased the assay sensitivity to 0.6 μg/mL relative to the original culture.

We added an additional control relative to TSST-1 measurements. Pluronic L92 is a surfactant that has previously been shown to increase TSST-1 production by USA200 (CC30) strains of S. aureus at pluronic L92 concentrations of 10% in TH broth (52). Pluronic L92 was used in the current study, as in a previous study (52), as a positive control for TSST-1 production, with amounts well above the usual amount of TSST-1 produced in TH broth (4.8 μg/mL in the absence of pluronic L92 compared to 38.4 μg/mL in the presence of 10% pluronic L92). Here, the use of pluronic L92, which amplified TSST-1 production, emphasizes the need to ensure that products used on mucosal surface do not enhance TSST-1 production.

It is also important to note that the starting pH of the cultures was adjusted to 7.8. Pluronic L92 did not affect the growth of S. aureus strain MN8 used in this study; the stationary phase in the presence of pluronic L92 remained the expected 7.5× 10^9^ CFU/mL. However, there was an important difference in final pH between the two culture conditions: pH 6.2 ± 0.09 with S. aureus MN8 alone versus pH 7.8 ± 0.04 in the presence of pluronic L92 (Student’s *t* test, *P* < 0.001). TSST-1 was shown previously to be produced at pH of 6.5 to 9; lower pHs inhibited production (23). It is possible that the maintenance of pH above 6.5 accounted for the elevated TSST-1 production.

### HVECs.

The HVEC culture has been described previously (29). Briefly, the gently immortalized cells maintained the properties of the primary cells used to create them. The HVECs were cultured in keratinocyte serum-free medium until confluent in 96-well tissue culture plates. The cells were then used for experimentation. The cells were exposed to bacteria, as indicated in triplicate samples, for 6 h at 37°C, 5% CO_2_. Subsequently, the cells and bacteria were frozen at −20°C for 24 h. Next, IL-8 was measured using Quantikine kits from R&D Systems (Minneapolis, MN). We have previously shown that IL-8 is resistant to proteases produced by menstrual TSS S. aureus (32).

### Statistics.

Means ± standard deviations were determined. Student’s *t* test was used to determine significant differences between means.
